# Therapie von schwerbehandelbaren chronischen Wunden mit Hyaluronsäureester: Eine Fallbeschreibung über sechs Patient*innen

**DOI:** 10.1007/s10354-021-00831-z

**Published:** 2021-03-18

**Authors:** Emanuel Maitz, Barbara Binder

**Affiliations:** 1 Univ.-Klinik für Orthopädie und Traumatologie, Medizinische Universität Graz, Auenbruggerplatz 5, 8036 Graz, Österreich; 2 Univ.-Klinik für Dermatologie und Venerologie, Medizinische Universität Graz, Auenbruggerplatz 8, 8036 Graz, Österreich

**Keywords:** Hyaluronsäureester, Hyaluronsäureester-Flies, Chronische Wunden, Hyalofill®, Hyaff®, Hyaloronic acid ester, Hyaloronic acid ester fleeze, Chronic wounds, Hyalofill®, Hyaff®

## Abstract

Die Behandlung von chronischen Wunden ist meist eine große Herausforderung für alle Beteiligten. Oft ist der Weg bis zur Heilung langwierig und frustran, sodass verschiedene Therapieversuche durchgeführt werden müssen, bis eine Heilung erzielt wird. In dieser retrospektiven Fallbeschreibung berichten wir über den Behandlungserfolg durch die Therapie mit einem Hyaluronsäureester-Flies, den wir bei 6 Patient*innen mit chronischen Wunden unterschiedlicher Genese erzielen konnten. Alle Patient*innen bekamen zusätzlich Kompressionsbandagen oder Kompressionsstrümpfe, zwei zusätzlich eine Druckentlastung und alle wurden, wenn nötig, debridiert. Die chronischen Wunden von 5 der 6 Patient*innen heilten vollständig oder nur mit einem minimalen Restdefekt ab, 1 Patient war weiterhin therapieresistent. Speziell bei Patient*innen mit Grunderkrankungen, welche eine erfolgreiche Therapie besonders erschweren, konnten wir feststellen, dass durch eine Behandlung mit Hyaluronsäureestern dennoch gute Erfolge erzielt werden können.

## Abstract (en)

The treatment of chronic wounds is usually a major challenge for the involved medical staff. Various different treatment options have to be tested achieving a satisfactory result. In this retrospective case report, we describe successful treatments with hyaluronan acid ester fleece in six patients with chronic wounds of different origins. Furthermore, all patients received compression bandages or compression stockings, two patients were treated additionally with pressure relief devices and all were debrided if necessary. The chronic wounds of five out of the six patients healed completely over a period of 1.5–11 months or only with a minimal residual defect. However, one patient was still resistant to therapy over five months. In summary, the treatment with hyaluronan esters achieved good results especially in patients with complex medical history that makes a successful therapy particularly difficult.

## Einleitung

Über eine allgemein gültige Definition von chronischen Wunden wird noch diskutiert [1]. Jedoch hat die Deutsche Gesellschaft für Wundheilung und Wundbehandlung in der S3-Leitlinie mit Stand 2012 eine Wunde als chronisch definiert, wenn die Wunde mit einem Integritätsverlust der Haut und einer oder mehrerer darunter liegenden Strukturen einhergeht und nicht innerhalb von acht Wochen abheilt [2]. Aufgrund der unterschiedlichen Definitionen ist es schwierig, eine exakte Prävalenz anzugeben. [2–4].

2016 wurde von Heyer et al. eine Analyse der chronischen Wunden in Deutschland veröffentlicht, die zeigen konnte, dass chronische Ulzerationen am Unterschenkel (venöse, arterielle, gemischte und nicht spezifierte Ulcera) mit einer Prävalenz von 0,70 % die häufigste, und diabetische Fußulcera mit 0,27 % die zweithäufigste Ursache sind; daneben Druckulcera mit 0,18 % und Ulcera anderer Entitäten wie Pyoderma gangrenosum und Gangrän mit 0,03 % [3]. Die Lebensqualität wird durch diese chronischen Wunden, inklusive sozialer Ausgrenzung, negativ beeinflusst [2, 5–8]. Hinzu kommt der große therapeutische und pflegerische Aufwand, der mit hohen Kosten für das Gesundheitssystem und die Gesellschaft allgemein einhergeht [9–11]. Tritt eine Stagnation während der Behandlung auf, wird die Situation noch zusätzlich verschlechtert. Die Wahl des entsprechenden lokaltherapeutischen Produktes kann eine therapeutische Herausforderung darstellen [1, 12]. Hyaluronsäure-haltige Wundauflagen, in verschiedenen Formen angeboten, können in solchen Fällen die Wundheilung positiv beeinflussen [13].

Hyaluronsäure oder auch Hyaluronan (HA) ist der Hauptbestandteil der extrazellulären Matrix. Es ist ein Glykosaminoglykan, also ein langkettiges, lineares Polysaccharid mit einem Molekulargewicht von 105 bis 107 Dalton. Es ist unter anderem Hauptbestandteil des Gallertkerns der Bandscheiben, der Synovia und des Glaskörpers [1, 13, 14]. Als Teil der extrazellulären Matrix hat es einen entscheidenden Anteil an allen Prozessen in der Wundheilung. Es stimuliert die epitheliale Zellproliferation und -migration, weiters steigert es die Bildung von Granulationsgewebe und reduziert die Entzündungsreaktion [15, 16].

Mit den folgenden Fallbeschreibungen wollen wir die Wirksamkeit der Behandlung mit einem Hyaluronsäureester-Flies bei therapieresistenten chronischen Wunden darstellen.

## Material, Methoden, Ergebnisse

Es wurden 6 Patient*innen im Alter zwischen 38 und 83 Jahren retrospektiv beschrieben, die im Zeitraum von April 2015 bis August 2017 in unserer dermatologischen Gefäßambulanz mit Hyaluronsäureester-Flies behandelt wurden (EK-Nummer: 30-277 ex 17/18). Als Datenquellen dienten die schriftlichen Ambulanzbefunde, Fotodokumentationen und Planimetrien.

Angewandt wurde Hyalofill®-F (Hyaff®/absorbierende Hautmatrix; der Firma Anika Therapeutics, Padua, Italien), ein 100 % Ester der Hyaluronsäure entsprechend den Anwendungshinweisen des Erzeugers. Als Sekundärverband wurde ein Polyurethanschaumstoff appliziert.

Eingeschlossen wurden 2 Frauen und 4 Männer im Alter zwischen 38 und 83 Jahren mit chronischen Ulcera an den Unterschenkeln oder Füßen, welche seit mindestens 4 Monaten bestanden. Die zu Grunde liegenden Ursachen der Ulcera waren dabei unterschiedlich: 3 venöse Ulcera, 1 diabetisches Fußsyndrom, 1 Ulcus bei peripherer Polyneuropathie und 1 posttraumatisches Ulcus (Tab. 1). Bei jedem Patienten kam das Hyalofill®-F in der Granulations- und Epithelisationsphase zum Einsatz. Vor Behandlungsbeginn wurde bei allen Patient*innen eine ausführliche Anamnese inkl. Begleiterkrankungen erhoben, ebenso die aktuelle Medikation und Allergieanamnese. Laboruntersuchungen fanden nicht statt. Ein arterieller Doppler wurde durchgeführt und der arterielle Dopplerindex (ABI) berechnet. Bei allen beschriebenen Patient*innen war der ABI > 1, sodass eine klinisch relevante arterielle Durchblutungsstörung ausgeschlossen werden konnte. Regelmäßige Verbandswechsel (2–3 ×/Woche) durch Hauskrankenpflege, Patient*innen oder deren Angehörige bzw. beim Allgemeinmediziner und ärztliche Kontrollen in unserer Ambulanz (Patientin 4 anfangs 1 ×/Woche, ansonsten alle 2–4 Wochen) wurden vor, während und nach dem Beobachtungszeitraum durchgeführt. Alle Patient*innen bekamen Kompressionsbandagen oder Kompressionsstrümpfe und wurden, wenn nötig, chirurgisch debridiert (mit dem scharfen Löffel oder einer Ringkürette nach vorheriger Oberflächenanästhesie mit einem Lidocain-Spray). Zusätzlich erfolgte eine Druckentlastung bei Patient 2 und 5 mittels orthopädischem Verbandsschuh; ansonsten wurde keine zusätzliche Begleittherapie parallel durchgeführt.

**Table 1:** 

Patient	Lokalisation	Art	Seit wann bestehend	Größe am Beginn der Behandlung	Behandlungszeit mit Hyaluronsäureester	Abgeheilt
Patient 1	Innenknöchel rechts	Ulcus cruris venosum	27 Monate	3,8 × 0,8 cm	11 Monate	Ja
Patient 2	Großzehenballen links	Diabetisches Fußsyndrom und venöse Insuffizienz	6 Monate	1,5 cm lang	7 Monate	Ja
Patientin 3	Äußerer Unterschenkel rechts	Ulcus cruris venosum	34 Monate	2 × 2 cm	4 Monate	Ja, nach weiteren 2 Monaten
Patientin 4	Unterschenkel rechts	Traumatisches Ulcus, Varicosis	4 Monate	3,5 × 4 cm	1,5 Monate	Ja
Patient 5	Großzehenballen rechts	Malum perforans	14 Monate	3 × 2 cm	5 Monate	Restdefekt (3 mm lang, 3 mm tief)
Patient 6	Innenknöchel links	Ulcus cruris venosum	>10 Jahre	3 × 5,5 cm	5 Monate	Nein, keine Besserung

### Patient*innendetails

Pat 1: 77a, männlich, Raucher seit 40 Jahren, venöses Ulcus cruris am rechten Innenknöchel bestehend über 27 Monate. Der Patient war bereits mit verschiedenen Lokaltherapien behandelt worden; zuletzt durchgeführte Lokaltherapie: Hydrofaserverband. Nach elf Monaten Behandlung mit Hyaluronsäureester heilte das Ulcus vollständig ab.

**Figure 1:**
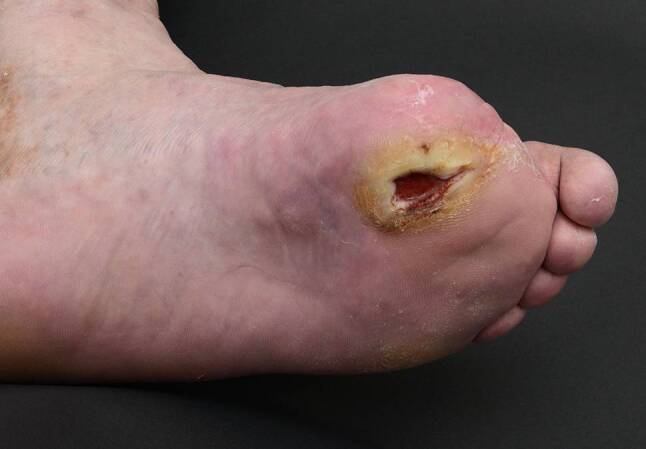

Pat 2: 60a, männlich, 2005 wurde die linke Großzehe wegen einer Osteomyelitis amputiert. Bevor er mit Hyaluronsäureester behandelt wurde, bestand seit sechs Monaten ein tiefes, spaltförmiges Ulcus an der Amputationsstelle. Der Patient hatte sich selbst therapiert. Zuletzt durchgeführte Lokaltherapie: Silbersulfdiazin-Gel und Schaumstoff. Sieben Monate nach Beginn der Hyaluronsäureesterbehandlung heilte dieses ab, (Abb. 1 und 2).

**Figure 2:**
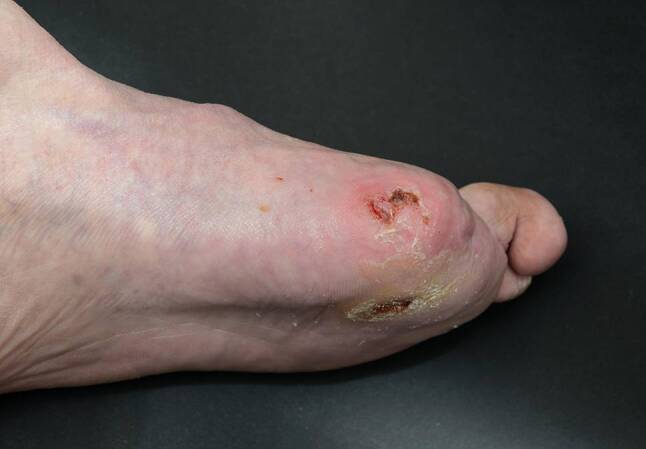

Pat 3: 83a, weiblich, seit 34 Monaten bestehendes Ulcus cruris venosum am rechten Außenknöchel. Zuletzt durchgeführte Lokaltherapie: Medizinischer Honig, Hydrofaserverband und Superabsorber. Nach vier Monaten Therapie mit Hyaluronsäureester, wurde auf Wunsch der Patientin wegen zeitweiser Schmerzen der Hyaluronsäureester abgesetzt und es erfolgte anschließend drei Monate eine Therapie mit dem bisher verwendeten Polyurethanschaumstoffverband mit vollständiger Abheilung.

**Figure 3:**
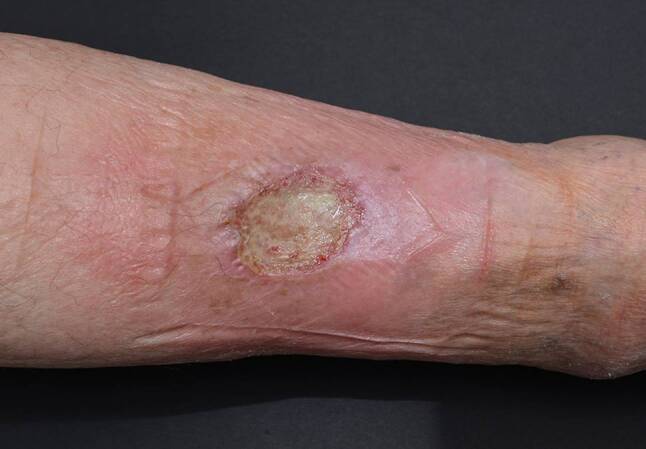

Pat 4: 76a, weiblich, mit einem traumatisch bedingten Ulcus cruris rechts, durch eine unbehandelte Varicosis begünstigt, welcher sich etwa vier Monate in einem therapieresistenten Zustand befand. Die bisherige Therapie wurde extern durchgeführt ohne entsprechende Kompressionstherapie. Zuletzt durchgeführte Lokaltherapie: Hydrofaserverband+Silber und Schaumstoff+Silber. Eine Abheilung konnte nach eineinhalb Monaten Therapie mit Hyaluronsäureester erzielt werden (Abb. 3 und 4).

**Figure 4:**
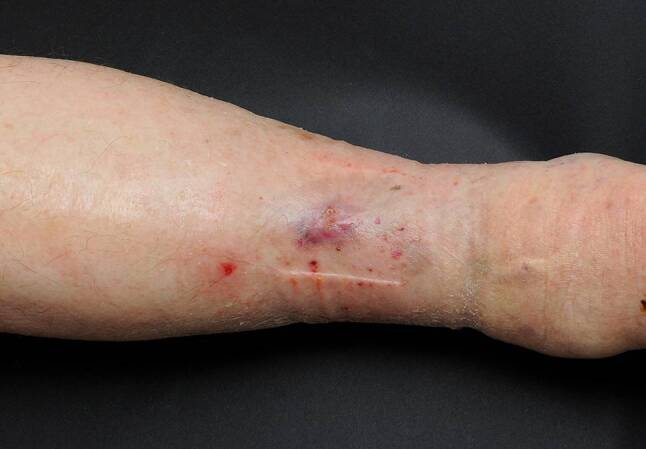

Pat 5: 38a, männlich, mit Malum perforans am rechten Großzehenballen (seit über einem Jahr), mit Sondierung bis zum Knochen (eine Osteomyelitis wurde radiologisch ausgeschlossen), bedingt durch ein diabetisches Fußsyndrom. Zuletzt durchgeführte Lokaltherapie: Hydrofaserverband+Silber. Es wurde fünf Monate mit Hyaluronsäureester behandelt, bis eine weitgehende Abheilung mit einem 3 mm langem und 3 mm tiefem Restdefekt, erzielt werden konnte.

Pat 6: 64a, männlich, mit Ulcus cruris venosum, bestehend seit mehr als 10 Jahren, konnte auch nach fünf Monaten Therapie mit Hyaluronsäureester keine deutliche Befundbesserung erreicht werden. Zuletzt durchgeführte Lokaltherapie: polyabsorbierendes Polyacrylat. Die Wundheilung wurde durch eine Adipositas permagna und latente cardiale Dekompensation, wobei die Kompressionstherapie ausgesetzt werden musste, negativ beeinflusst.

## Diskussion

Speziell behandlungsresistente chronische Ulcera sind oft, sowohl für den Patienten/die Patientin und dessen/deren Angehörige, als auch für die behandelnden Mediziner*innen und die Pflege, ein großes und nicht selten herausforderndes Problem. In vielen Fällen ist nicht ersichtlich, warum keine Abheilung trotz phasengerechter Therapie erzielt werden kann. Mit Hyaluronsäureester konnten wir eine Verbesserung bis hin zur Abheilung erzielen. 4 von 6 heilten vollständig ab, Patient 5 zeigte nach 5 Monaten einen schlitzförmigen, 3 mm tiefen Restdefekt und kam nicht mehr zum weiteren Follow-up. Bei Patient 6 zeigte sich ein frustraner Therapieversuch aufgrund seiner Begleiterkrankungen und daher inkonsequenten Kompressionstherapie. Durch das angewandte Hyaluronsäureester-Flies konnten bei oben beschriebenen Patient*innen die Wunden aus der Stagnation befreit werden und der Heilungsprozess einsetzen. Limitierend in dieser Fallübersicht ist sicher die geringe Patient*innenzahl und die retrospektive Analyse der Daten. Außerdem ist das Resultat bei der Behandlung chronischer Wunden immer stark von der individuellen Compliance der Patient*innen abhängig und damit nur bedingt vergleichbar.

Einige Studien und Fallbeschreibungen konnten die Wirksamkeit von Hyaluronsäureestern bereits bestätigen [13–22]. Speziell bei Patient*innen mit Grunderkrankungen, die eine erfolgreiche Therapie besonders erschweren, zeigen diese, dass durch eine Behandlung mit Hyaluronsäureestern dennoch gute Erfolge erzielt werden können [13–18]. Eine Metaanalyse von Voigt und Driver untersuchte den Behandlungserfolg mit Hyaluronsäure bei Verbrennungen, chirurgischen epithelialen Wunden und chronischen Wunden, wobei sie aufzeigen konnten, dass bei 8 von 9 Studien ein signifikanter Erfolg erzielt werden konnte [23].

## Zusammenfassung

Mit den dargestellten Fallbeschreibungen konnte gezeigt werden, dass mit diesem Hyaluronsäureester-Flies eine Abheilung bzw. Verbesserung der Wundheilung bei therapieresistenten chronischen Wunden erzielt werden kann. Dennoch muss berücksichtigt werden, dass eine gewisse Behandlungszeit und Patient*innenmitarbeit nötig ist, um einen gewünschten Therapieerfolg zu erzielen. Hyaluronsäureester stellen jedoch eine zielführende Therapieoption für chronische Wunden dar, welche in der Heilung stagnieren und keine oder nur geringe Besserung unter der bisherigen Lokaltherapie zeigen. Randomisiert-kontrollierte, prospektive klinische Studien sind notwendig, um die Vorteile gegenüber bisher verwendeten Lokaltherapeutika empirisch zu belegen.
